# A comparative analysis of patient participation in health technology assessment systems worldwide: trends and practices

**DOI:** 10.3389/fpubh.2025.1693886

**Published:** 2025-10-31

**Authors:** Marcos Puebla, Luis Korrodi-Gregório, Luca Trentin, Annabel De Maria, Nerea Blanqué

**Affiliations:** ^1^Alira Health SLU, Barcelona, Spain; ^2^Alira Health Srl, Milan, Italy

**Keywords:** health technology, health technology assessment (HTA), patient engagement (PE), patient involvement, public involvement, patient participation

## Abstract

**Introduction:**

Over the last decades, Health Technology Assessment (HTA) has become pivotal in guiding decision-making concerning the inclusion of new health technologies in health systems worldwide. As HTA continues to evolve, the importance of patient participation to achieve a more informed, transparent, and legitimate decision-making process has gained increased recognition. Yet practices vary widely and are often modest in scope.

**Methods:**

We conducted a comparison and ranking of 56 HTA systems across five regions based on levels of patient participation throughout the HTA process. Participation was measured by applying a scoring framework we developed to publicly available information for each system.

**Results:**

Many HTA systems include patient participation, but the level of involvement shows substantial variation across systems and tends to be comparatively modest. Some systems demonstrate active engagement throughout the process, while others show limited participation.

**Discussion:**

To our knowledge, this is the first attempt to quantify patient participation on a wider scale to enable comparisons across a large sample of HTA systems. The findings offer policymakers, healthcare professionals, patients, and researchers a comprehensive view of current approaches and highlight opportunities to enhance patient involvement in HTA.

## 1 Introduction

Healthcare systems have limited resources and often have to make difficult decisions regarding the allocation of these resources in the service of providing effective, valuable, and timely care in an equitable manner (1). These decisions are complex and require a clear, comprehensive, and transparent process to ensure that they are fair and are acceptable to the population served. Health technology assessment (HTA), a multidisciplinary process that systematically summarizes and evaluates the medical, economic, social, and ethical issues related to the use of a health technology, is one of the most widely used methods for informing the decision-making process (2, 3).

As the role and methods of HTA continue to evolve, patient participation in HTA has emerged as imperative (4). In this article, “patients” is used as an umbrella term to encompass various roles representing patients, such as individual patients, caregivers, patient organization representatives, patient advocates, or patient experts. These roles are integral for making more informed, transparent, accountable, and legitimate decisions regarding health technologies. Despite several worldwide initiatives aimed at involving patients in HTA, practices for patient participation remain limited in many regions across the world (5–8). Such limitations often stem from barriers including limited resources, insufficient familiarity of patients with technical processes, lack of standardized methodologies, and uncertainty about how to meaningfully integrate patient input. In many systems, participation remains sporadic or tokenistic rather than institutionalized (4, 9, 10).

One of the main arguments for involving patients in HTA is based on the rights of patients to participate in the planning and delivery of their healthcare. Since the HTA process typically determines the health services and procedures available to patients, it follows that patients have a right to be heard as part of the HTA process (11). Beyond respecting patients' rights, such engagement has the added value of building public trust in the health system processes and in increasing transparency (8). Patients can also provide valuable perspectives on their experiences, attitudes, beliefs, values, and expectations regarding health, illness, the impact of health technologies, and their utilization–information that is not obtainable from clinical trials or medical practitioners (12). Consequently, integrating patients into HTA processes is expected to result in the delivery of care that is responsive to their unique needs and values (13). Beyond sharing their experiential knowledge, it is believed that involving patients in health decision-making can foster a sense of empowerment and contribute to more effective solutions when it comes to allocating limited healthcare resources (4). While evidence of the impact of patient participation on market access, whether in terms of accelerating availability or broadening access, remains limited and more time and systematic evaluation will be needed to fully understand its overall effects, there are documented cases where it has influenced HTA recommendations, such as the Patient and Clinician Engagement (PACE) process in Scotland (14), patient evidence shaping appraisal outcomes at NICE in the United Kingdom (15), and patient submissions and consultations contributing to reimbursement decisions at CONITEC in Brazil (16). Ultimately, incorporating patient perspectives ensures that the specific requirements and values of patients are considered, enhancing both the legitimacy and the relevance of HTA decisions (9).

**Figure 1:**
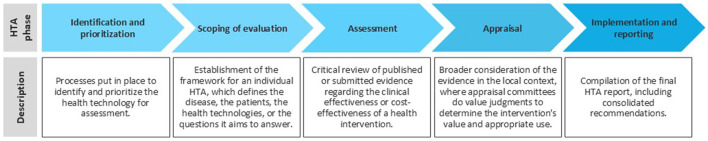
Description of HTA phases.

As outlined by Facey (17), patient involvement in HTA can be understood through two distinct but complementary approaches: patient-based evidence and patient participation. Patient-based evidence refers to data systematically generated through research, such as studies on experiences from patients, preferences, and outcomes, usually published in peer-reviewed literature. By contrast, patient participation draws directly on the perspectives and lived experiences of individual patients, caregivers, or patient groups, without necessarily following formal research methods. While both approaches enrich the HTA process, this article focuses specifically on patient participation mechanisms. As described in Figure 1, patient participation can occur at every step of the HTA process, from topic selection and scoping to assessment, appraisal, and implementation and reporting. Within these phases, participation can take different forms depending on the purpose and context, such as identifying and prioritizing health technologies, submitting input about a disease or health technology individually or through patient groups, reviewing HTA documentation, or serving as patient representatives on HTA committees (8, 17).

While there is already a notable acknowledgement of the relevance of patient participation in the HTA process, the breadth and depth of that participation can vary significantly from one country to another, or even across HTA systems in the same country. It can still be perceived as an add-on rather than an indispensable component; hence, its absence is not necessarily viewed as a critical deficiency in an HTA, and there is still room for improvement (5–8).

Given the degree of heterogeneity present in both HTA systems themselves and the amount and type of patient participation observed in their processes, it can be difficult to make comparisons across systems (17). Attempts have been made to o classify patient participation in HTA (18), as well as to describe and catalogue the role of patients in HTA in individual countries and there are various published studies comparing a particular activity or set of practices between countries or across groups of countries (19–26).

However, to the best of our knowledge, the present study is the first attempt to systematize and, in particular, quantify, the level of participation on a wider scale to enable comparisons across countries and geographies and recognize leaders and areas for improvement. Previous studies have been primarily descriptive, while this study, in developing a weighted index, aims to quantify participation to allow for a more robust comparison across HTA systems and capture a snapshot of the current state of affairs, that can be used as a baseline against which future developments can be measured.

## 2 Materials and methods

### 2.1 Scope and variables definition

Prior to identifying or evaluating any HTA systems, the scope of HTA and the set of activities in which patient participation was deemed to be important were established. The list was based on published literature (4, 10, 17–19, 21, 27–30) and was designed to capture a comprehensive yet manageable set of recognized practices, spanning all HTA phases and balancing structural mechanisms (e.g., voting rights, committee membership), procedural practices (e.g., consultations, submissions, report reviews), and transparency measures (e.g., lay summaries, feedback on input use). The phases of HTA that were considered in scope for the purposes of this study were identification and prioritization of health technologies, scoping of evaluation, assessment, appraisal, and implementation and reporting, which parallel those defined by Goodman (31). Within each HTA phase, relevant activities were defined as variables, and a set of categories were developed for each variable to measure the level of patient participation. Additionally, a few variables were independent of these phases and applied to the overall HTA process. Table 1 presents the 17 variables and their main categories used to assess patient participation in the study. Three of the 17 variables were not classified within the phases due to their application to the general HTA process. A more detailed breakdown of the categories and variables can be found in Supplementary Table 1.

**Table 1:** Patient participation variables and categories.

**HTA process**
**Geographic scope of HTA system**
Regional scope
National scope
**Establish initiatives to build capacity for patients to contribute**
Not implemented
Some grade of capacity building
Incorporates at least three capacity-building activities^*^ or a combination of support team and training
**Establish initiatives to self-evaluate and improve patient participation**
Not implemented
Some level of self-evaluation, usually by the HTA system asking for feedback and advice from patients
Incorporates a self-evaluation process, or has conducted at least one self-evaluation, or has a team to enhance participation
**Individual HTA**
**Phase I: identification and prioritization**
***Allow patients to participate in the identification and/or prioritization of health technologies***
Not implemented
Patients participate in the identification or prioritization
Patients participate in the identification and prioritization
**Phase II: scope of evaluation**
***Allow patients to participate in the scoping protocol development or review***
Not implemented
Some level of participation, but mechanism is unclear
Patients provide submissions to develop assessment/scoping protocol, or review the protocol
Patients participate as part of the scoping team or working groups/workshops that develop or review the protocol
**Phase III: assessment**
***Collect patients' perspectives through submissions or direct consultations***
Not implemented
Open call for anyone to submit information
Submissions or consultations of patient/consumer groups or selected patients
Submissions or consultations of patient/consumer groups or selected patients, and patient experts' statements
***Allow patients to participate at assessment meetings, technical tables, or working groups***
Not implemented
Patients participate as non-members or in working groups
Patients participate as members of the assessment team
***Allow patients to review the assessment report or improve the patient input***
Not implemented
Mechanisms exist, but these are unclear and non-routinely applied
Allow patients to review the assessment report or improve the patient input before the committee meeting
**Phase IV: appraisal**
***Present the patient submissions or testimonies to the appraisal committee***
Not implemented
Patients attend as observers, or the mechanism of participation is very unclear
Patients can attend and participate, or their input is presented, but the mechanism is unclear or non-routinely applied
Patient input is routinely presented by non-input contributors or non-spokepersons
Patient input is routinely presented by direct contributors or spokepersons
***Include patients/consumers/public as members of appraisal committees***
Not implemented
Citizens or citizens representatives, or public members (representing general public)
Patients/consumers, or patient/consumer representatives, or lay members/public members (if they act as patient representatives)
***Grant patient/public/consumer members the right to vote***
Not implemented
Yes
***Allow patients to review the draft recommendation report***
Not implemented
Mechanisms exist, but these are unclear and non-routinely applied
Clear mechanism and routinely applied
***Hold committee meetings fully or partially in public***
Not implemented
Yes
**Phase V: implementation and reporting**
***Allow patients to participate in the appeal process***
Not implemented
Patients participate, but mechanism is unclear, or non-routinely applied, or patients act only as observers of the final deliberation
Either starting the appealing process or actively impacting on the outcome
***Produce easy to read summaries of HTA reports***
Not implemented
Yes
***Include patient input in the HTA reports/recommendations***
Not implemented
Assessment report includes patient input
HTA recommendations acknowledge or mention the patient input, but no summary is included
HTA recommendations include a summary of the patient input
***Summarize/inform how patient input was used and impacted the recommendations***
Not implemented
The report with recommendations mentions how patient input was used and impacted the recommendations/decisions

^*^Providing training, patient participation guidance, a support team for involvement, information about the technology, or a template for submissions.

These formed the components of a scoring system (range 0–10), in which activities were weighted based on their significance to the HTA process and outcome and labelled as *Low, Medium, High*, or *Very High* relevance. Weights were determined using a simplified three-factor framework that considered: (i) the depth and role of engagement (symbolic, consultative, or empowered), (ii) the influence the activity can exert on HTA outputs, and (iii) its contribution to transparency or the institutionalization of patient participation. Activities that embedded patients structurally or granted decision-making power (e.g., voting rights, committee membership) were assigned *Very High* weights, whereas activities offering active but non-structural participation (e.g., assessment meetings, scoping, patient testimonies) were assigned *High* weights. *Medium* weights were applied to activities that support participation or enhance transparency without strong influence (e.g., self-evaluation, draft review, public meetings), while *Low* weights reflected activities that were largely symbolic or informative (e.g., lay summaries, report mentions). The full table detailing weight assignments and corresponding rationales is provided in Supplementary Table 2.

Each activity was assessed using a 0–1 scoring system to capture the level of implementation across HTA agencies. For every activity, predefined categories described graduated levels of patient participation, ranging from 0 (not implemented) to 1 (highest level of participation), with partial scores (e.g., 0.2, 0.4, 0.5, 0.6, 0.8) reflecting intermediate levels. Partial points were determined by activity-specific criteria, including the clarity and formalization of the participation mechanism, frequency of its application (routine vs. non-routine), and type of participants involved (with higher scores for direct patient or patient-organization involvement compared to general public representation). This approach aligns with the paper's primary focus on patient participation, although public participation was also considered, recognizing its potential to convey the patient perspective in certain contexts. The term “patient participation” is used throughout the text to refer to both patient and public participation, unless otherwise specified. By combining flexibility with comparability, this scoring system captures the nuanced variability of implementation within and across HTA systems while maintaining comparability. The complete list of activity-specific categories and their corresponding scores is provided in Supplementary Table 3. Total HTA system scores were calculated by summing the weighted activity scores, yielding a final score between 0 and 10.

### 2.2 Country selection

In the first phase of the process of identifying and evaluating HTA systems, countries for inclusion in the analysis were selected. Firstly, through a scouting exercise, countries with formal HTA systems were identified and evaluated for inclusion in the analysis. Starting from the list of United Nations member states (plus Taiwan), each country was cross-referenced with various publicly available lists of HTA systems (INAHTA, EUnetHTA, WHO, publications, reports, etc.) to see if there were any formal HTA body present in the country. In addition, for each country, the Human Development Index (HDI) category and gross domestic product (GDP) were recorded.

Once the check for HTA systems was complete and the HDI and GDP information had been collected, the country selection process began. Countries were selected for inclusion based on the presence of HTA systems, as well as their HDI category and GDP. Countries without at least one formal HTA body were excluded from the study. Next, a filter for HDI category was applied, in which those countries with a very high HDI (all countries meeting this criterion) or high/medium HDI (and with a GDP above 250 million to ensure representativeness of countries) were included. Then a GDP cut-off was applied to the remaining countries, with only those countries whose GDP was above 200 million USD advancing to the next stage. In the final stage of the selection process, the type of the HTA body was evaluated. Countries with at least one entity that was an agency, committee, or similar, and that had a web presence were included, while those whose HTA systems were units, directorates, or technical groups within the country's Ministry of Health, social security organization, national commission, or medicines agency, or who lacked a web presence, were excluded. The rationale for this selection phase was to be judicious and follow a process that could be replicated in the future and at the same time ensure that there would be sufficient information available on each country for the evaluation phase.

### 2.3 HTA system selection

In the second phase, following the conclusion of the country selection process, further information was collected about the HTA systems in the remaining countries to determine which HTA systems from these countries would be analyzed in the study. The HTA systems in each country were identified and basic information (name, geographic scope, remit, website, etc.) was compiled about each of the systems.

HTA systems with a national scope were preferentially identified. However, in certain cases, such as when HTA was also taking place at a sub-national level, a few regional systems were also included. If a particular nation had more than one HTA body with a national scope, with one for pharmaceuticals and another for medical devices, for example, or one for inpatient and another for outpatient drugs, then multiple systems were included. Systems responsible for HTA of pharmaceuticals, medical devices, or both were included, while systems that exclusively focused on medical procedures or diagnostics were excluded. In addition, when more than one national HTA body evaluated pharmaceuticals and/or medical devices, only the body whose assessments inform decision-making regarding the inclusion and coverage was included. Within the HTA systems that inform decision-making, processes or programs not related to coverage, such as multiple technology assessment (MTA) in France or the development of clinical guidelines in Colombia, were not included. There was an emphasis on public systems performing HTA activities related to public healthcare systems, with the exception of the United States and Brazil, where private activity was also considered. This exception was made because, unlike in most countries, the United States and Brazil do not rely on a single national public HTA system. In the US, where coverage decisions are decentralized across public and private payers, influential private HTA bodies such as ICER complement the work of public agencies like AHRQ and directly shape reimbursement practices. Similarly, in Brazil, the health ecosystem includes both the public SUS (CONITEC) and a large regulated private sector where COSAUDE conducts HTA.

### 2.4 Data collection and analysis

The third phase was data identification and extraction. For each HTA agency selected in phase two searches were performed to identify additional sources of relevant information, after which data on the various facets of patient participation in the HTA process were extracted from the publicly available information into a spreadsheet. Triangulation of three data sources was undertaken to support data verification: sources of information primarily included: official HTA organization websites (identified during the scouting phase), published research papers, and grey literature (e.g., government or international organization reports). In all cases, the HTA body's own website as well as other official national or regional websites with information about the workings of that particular entity and its processes were reviewed in detail. Relevant published papers were identified via PubMed and manual searching of the references in already identified publications or reports. Translation tools were used as needed. Only secondary/desk research was conducted—the HTA systems were not contacted.

Data extraction for each agency was performed by a single reviewer, who filled in the spreadsheet with the available information, as well as the source or sources for each data point. Using this information, the reviewer then followed a rubric to assign a number for each data point that represented the degree to which each of the pre-identified criteria were fulfilled. A second reader then reviewed both the information collected for each HTA body and the degree of fulfillment assigned by the first reviewer in order to ensure accuracy and consistency in the application of the rubric across all systems evaluated. Discrepancies were resolved by consensus, with a third reviewer acting as referee if necessary.

After completing data extraction and application of the rubric, the sub-section (variable level), section (HTA phase level), and overall scores (0–10) were calculated for each HTA body, which can be found in Supplementary Table 4. The three variables that were not assigned to one of the four HTA phases only counted individually and for the overall score. These scores were then compared to each other and the HTA systems ranked accordingly. A qualitative analysis was also performed to explore trends, describe best practices, and identify the areas where patient participation was most lacking.

## 3 Results

In the first phase of country selection, out of the 194 countries considered for inclusion in the universe of the study, one or more formal HTA systems were detected during the scouting phase in 88 countries, with the remaining 106 excluded at this stage. In the second phase, a further 27 and 19 were excluded based on HDI and GDP, respectively, leaving 42 countries. Finally, an additional five countries were excluded based on the type of HTA organization or because the identified HTA systems lacked websites, with the result that a total of 37 countries were included in the analysis.

In the third phase, from the 37 countries selected, a total of 56 HTA systems were included in the analysis. Out of the 56, 29 HTA systems were in Europe, 12 in Asia, seven in North America, five in Latin America, and three in Oceania. No HTA systems from the region of Africa met the filtering criteria for inclusion in the analysis. Only a single HTA body was evaluated in 25 countries, two systems were evaluated in seven countries, three systems in three countries, and four systems in two countries. Twelve HTA systems were exclusively dedicated to the HTA of pharmaceuticals, 12 exclusively to medical devices, and 32 covered both. Eight countries had separate systems for drugs vs. medical devices. Forty-nine HTA systems were national in their geographic scope, while seven systems were regional. The full list of HTA systems and further details can be found in Table 2.

**Table 2:** Distribution per region, geographic scope and remit of the final list of analyzed HTA systems.

**HTA system**	**Full name**	**Country**	**Geographic scope**	**Remit**
**Europe (29 HTA systems)**				
AEMPS (48, 49)	Spanish agency for medicines and medical devices	Spain	National	PH
AGENAS (17, 50, 51)	Italian national agency for regional healthcare services	Italy	National	MD
AIFA (51–54)	Italian medicines agency	Italy	National	PH
AIHTA (40, 55, 56)	Austrian institute for health technology assessment	Austria	National	MD
AOTMIT (20, 57–65)	Agency for health technology assessment and tariff system	Poland	National	PH and MD
CATSALUT (66–68)	Catalan health service	Spain	Regional	PH
CRUF (69, 70)	Regional single coordination on medicines	Italy	Regional	PH and MD
DHTC (71–73)	Danish health technology council	Denmark	National	MD
DMC (17, 74–80)	Danish medicines council	Denmark	National	PH
DVSV (81, 82)	Austrian social insurance	Austria	National	PH
FIMEA (83–88)	Finnish medicines agency	Finland	National	PH
FOPH (89–93)	Federal office of public health	Switzerland	National	PH and MD
IQWIG–G-BA (17, 94–101)	Institute for quality and efficiency in health care–federal joint committee	Germany	National	PH and MD
HAS (19, 20, 35, 102–115)	French national authority for health	France	National	PH and MD
HIQA (116–119)	Health information and quality authority	Ireland	National	MD
INFARMED (120–123)	National authority of medicines and health products	Portugal	National	PH and MD
KCE-RIZIV/INAMI (34, 124–127)	Belgian health care knowledge centre–national institute for health and disability insurance	Belgium	National	PH and MD
RETEHTA (70, 128)	Regional health technology assessment agency of Liguria	Italy	Regional	MD
NAMMDR (129–131)	National agency for medicines and medical devices of Romania	Romania	National	PH and MD
NCPE–HSE (132–137)	National centre for pharmacoeconomics–health service executive	Ireland	National	PH
NICE (15, 17, 20, 138–146)	National institute for health and care excellence	United Kingdom	National	PH and MD
NIPHNO (51, 147–159)	Norwegian institute of public health Norway	Norway	National	MD
NOMA (51, 149, 150, 152–155, 159–162)	Norwegian medicines agency	Norway	National	PH
REDETS (163)	Spanish network of agencies for assessing national health system technologies and performance	Spain	National	MD
SHTG (17, 23, 164–170)	Scottish health technologies group	United Kingdom	National	MD
SMC (17, 20, 146, 171–178)	Scottish medicines consortium	United Kingdom	National	PH
SÚKL (34, 129, 158, 179, 180)	State INSTITUTE FOR DRUG CONtrol	Czech Republic	National	PH
TLV (17, 159, 181)	Dental and pharmaceutical benefits agency	Sweden	National	PH and MD
ZIN (19–21, 182–185)	National health care institute	Netherlands	National	PH and MD
**Asia (12 HTA systems)**				
ACE (186–192)	Agency for care effectiveness	Singapore	National	PH and MD
C2H (6, 186)	Center for outcomes research and economic evaluation for health	Japan	National	PH and MD
CNHDRC (193–195)	China national health development research center	China	National	PH and MD
DHR-HTAIn (196)	Directorate of health research–regional resource centre for health technology assessment in India	India	National	PH and MD
HIRA (6, 22, 197)	Health insurance review and assessment service	Republic of Korea	National	PH
HITAP (46, 186, 198–202)	Health intervention and technology assessment program	Thailand	National	PH and MD
HSPI (186, 203–205)	Health strategy and policy institute	Vietnam	National	PH and MD
HTAC (186, 206, 207)	Health technology assessment council	Philippines	National	PH and MD
ICTAHC (208–211)	Israeli center for technology assessment in health care	Israel	National	PH and MD
MAHTAS (212, 213)	Malaysian health technology assessment section	Malaysia	National	PH and MD
NECA (6, 36, 214–219)	National evidence-based healthcare collaborating agency	Republic of Korea	National	MD
NIHTA (17, 186, 214, 220–222)	National institute of health technology assessment	Taiwan	National	PH and MD
**North America (seven HTA systems)**				
AHRQ (19, 223–227)	Agency for healthcare research and quality	United States	National	PH and MD
CDA-AMC (17, 228–234)	Canada's drug agency	Canada	National	PH and MD
ICER (235–242)	Institute for clinical and economic review	United States	National	PH and MD
INESSS (17, 44, 243)	National institute of excellence in health and social services	Canada	Regional	PH and MD
OHA-HERC (19, 244, 245)	Oregon health authority–health evidence review commission	United States	Regional	MD
OHTAC (17, 246–249)	Ontario health technology advisory committee	Canada	Regional	PH and MD
WSHCA-HTCC (19, 250)	Washington state health care authority–health technology clinical committee	United States	Regional	MD
**Latin America (five HTA systems)**				
CENETEC (41, 251)	National center for health technology excellence	Mexico	National	PH and MD
CONETEC (252–258)	National commission for health technology assessment and clinical excellence	Argentina	National	PH and MD
CONITEC (17, 41, 42, 44, 259–264)	National committee for technology incorporation	Brazil	National	PH and MD
COSAUDE (42, 265)	Permanent healthcare regulation committee	Brazil	National	PH and MD
IETS (20, 41, 45, 266)	Institute for evaluation of health technologies	Colombia	National	PH and MD
**Oceania (three HTA systems)**				
MSAC (19, 267–276)	Medical services advisory committee	Australia	National	MD
PBAC (17, 19, 277–285)	Pharmaceutical benefits advisory committee	Australia	National	PH
PHARMAC (19, 286–295)	Pharmaceutical management agency	New Zealand	National	PH

MD, medical devices; PH, pharmaceuticals.

### 3.1 Phases and variables results: occurrence of patient participation by phase and variable across all HTA systems

**Figure 2:**
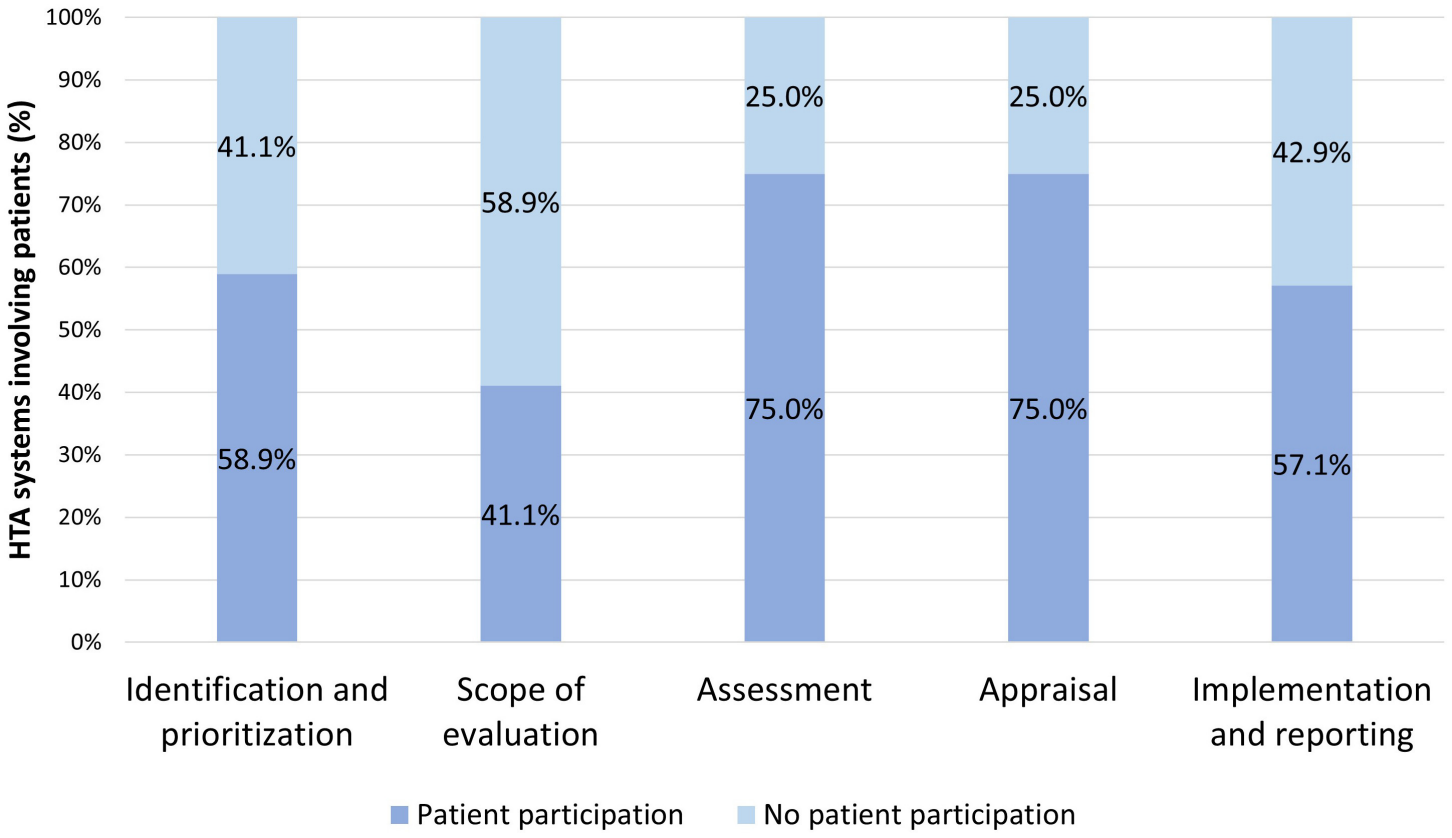
Occurrence of patient participation per phase across all HTA systems.

Figure 2 displays the occurrence of patient participation per phase across all HTA systems, without delving into the degree of participation. Notably, patient participation was most frequent in the assessment and appraisal phases, with the scoping phase exhibiting the least.

**Figure 3:**
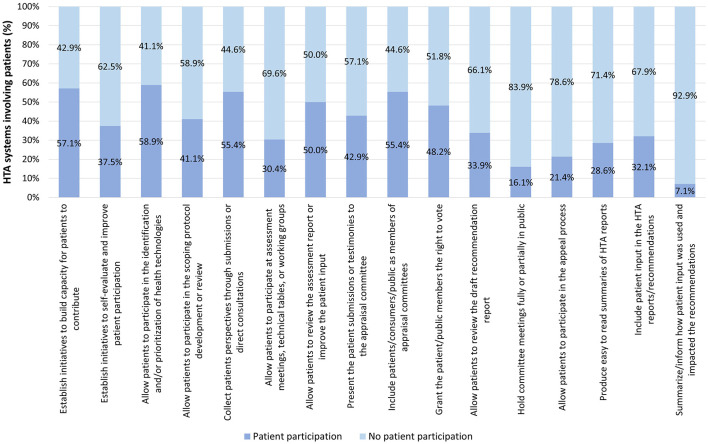
Occurrence of patient participation per variable across all HTA systems.

Figure 3 outlines the occurrence of patient participation per variable across all HTA systems, without considering the depth of participation per variable. Of the 16 variables used to evaluate patient participation -excluding the variable of “geographic scope of the HTA system” as greater emphasis was placed on those directly related to patient participation-, the three with the highest occurrence were, in descending order: allowing patients to identify and/or prioritize health technologies for evaluation (58.9% of HTA systems), building capacity for patients to contribute (57.1%), collecting patients' perspectives through patient submissions or direct consultation, and including patients/consumers/public as members of appraisal committees (55.4% for both). The three variables that least commonly included patient participation were, in ascending order: summarizing/informing how patient input was used and impacted the recommendations (7.1%), holding committee meetings in public (16.1%), and allowing patients to participate in the appeal process (21.4%).

### 3.2 Phases and variables results: deep dive into the comprehensiveness of patient participation across phases and their respective variables and categories in all HTA systems

Table 3 displays the distribution of patient participation by variable and category across all HTA systems, offering insight into the depth of the patient participation per variable, Additionally, it provides examples within each category to further illustrate the findings. The categories within the variables in this table have been simplified for clarity. The full, detailed table can be found in Supplementary Table 5.

**Table 3:** Distribution of patient participation by variable and category across all HTA systems.

**Variables and categories**	HTA systems (%^*^)	**Examples of HTA systems**
**HTA process**		
***Geographic scope of HTA system***		
Regional scope	12.5%	CATSALUT, CRUF, OHA-HERC
National scope	87.5%	CDA-AMC, FOPH, HTAC
***Establish initiatives to build capacity for patients to contribute***		
Not implemented	42.9%	CENETEC, DHR-HTAIn, SÚKL
Some grade of capacity building	26.8%	NECA, NOMA, DMC
Incorporates at least three capacity-building activities^**^ or a combination of support team and training	30.4%	SMC, CONITEC, CDA-AMC
***Establish initiatives to self-evaluate and improve patient participation***		
Not implemented	62.5%	IQWIG-G-BA, DVSV, NAMMDR
Some level of self-evaluation, usually by the HTA system asking for feedback and advice from patients	8.9%	ZIN, HIQA, SHTG
Incorporates a self-evaluation process, or has conducted at least one self-evaluation, or has a team to enhance participation	28.6%	MSAC, HAS, NICE
**Individual HTA**		
**Phase I: identification and prioritization**		
***Allow patients to participate in the identification and/or prioritization of health technologies***		
Not implemented	41.1%	SMC, NIHTA, AIFA
Patients participate in the identification or prioritization	41.1%	ZIN, PBAC, ICER
Patients participate in the identification and prioritization	17.9%	IQWIG-G-BA, NICE, CONITEC
**Phase II: scope of evaluation**		
***Allow patients to participate in the scoping protocol development or review***		
Not implemented	58.9%	ZIN, PHARMAC, HSPI
Some level of participation, but mechanism is unclear	7.1%	NOMA, NIPHNO, DHR-HTAIn
Patients provide submissions to develop assessment/scoping protocol, or review the protocol	16.1%	CDA-AMC, FOPH, HTAC
Patients participate as part of the scoping team or working groups/workshops that develop or review the protocol	17.9%	DMC, ICER, CONETEC
**Phase III: assessment**		
***Collect patient's perspectives through submissions or direct consultations***		
Not implemented	44.6%	AIHTA, MAHTAS, CRUF
Open call for anyone to submit information	23.2%	NIHTA, PBAC, AHRQ
Submissions or consultations of patient/consumer groups or selected patients	30.4%	SMC, IQWIG-G-BA, CDA-AMC
Submissions or consultations of patient/consumer groups or selected patients, and patient experts' statements	1.8%	NICE
***Allow patients to participate at assessment meetings, technical tables, or working groups***		
Not implemented	69.6%	FIMEA, HTAC, NCPE-HSE
Patients participate as non-members or in working groups	19.6%	IQWIG-G-BA, PHARMAC, NOMA
Patients participate as members of the assessment team	10.7%	CATSALUT, PBAC, DHTC
***Allow patients to review the assessment report or improve the patient input***		
Not implemented	50.0%	NCPE-HSE, NAMMDR, IETS
Mechanisms exist, but these are unclear and non-routinely applied	1.8%	HAS
Allow patients to review the assessment report or improve the patient input before the committee meeting	48.2%	AEMPS, AGENAS, FIMEA
**Phase IV: appraisal**		
***Present the patient submissions or testimonies to the appraisal committee***		
Not implemented	57.1%	ACE, HIQA, OHTAC
Patients attend as observers, or the mechanism of participation is very unclear	5.4%	CONETEC, MAHTAS, FIMEA
Patients can attend and participate, or their input is presented, but the mechanism is unclear or non-routinely applied	16.1%	AOTMIT, PHARMAC, HTAC
Patient input is routinely presented by non-input contributors or non-spokepersons	5.4%	HAS, DMC, DHTC
Patient input is routinely presented by direct contributors or spokepersons	16.1%	SMC, NIHTA, CONITEC
***Include patients/consumers/public as members of appraisal committees***		
Not implemented	44.6%	ACE, CONETEC, HITAP
Citizens or citizens representatives, or public members (representing general public)	7.1%	CONITEC, ICTAHC, HTAC
Patients/consumers, or patient/consumer representatives, or lay members/public members (if they act as patient representatives)	48.2%	AHRQ, PBAC, NECA
***Grant patient/public/consumer members the right to vote***		
Not implemented	51.8%	IQWIG-G-BA, C2H, NIHTA
Yes	48.2%	SMC, MSAC, DMC
***Allow patients to review the draft recommendation report***		
Not implemented	66.1%	PBAC, HAS, NECA
Mechanisms exist, but these are unclear and non-routinely applied	12.5%	MSAC, CONITEC, HTAC
Clear mechanism and routinely applied	21.4%	NICE, CDA-AMC, CONETEC
***Hold committee meetings fully or partially in public***		
Not implemented	83.9%	TLV, AEMPS, HAS
Yes	16.1%	ZIN, IQWIG-G-BA, ICER
**Phase V: implementation and reporting**		
***Allow patients to participate in the appeal process***		
Not implemented	78.6%	IETS, HITAP, DVSV
Patients participate, but mechanism is unclear, or non-routinely applied, or patients act only as observers of the final deliberation	14.3%	NOMA, NAMMDR, HIRA
Either starting the appealing process or actively impacting on the outcome	7.1%	NICE, IQWIG-G-BA, AHRQ
***Produce easy to read summaries of HTA reports***		
Not implemented	71.4%	CENETEC, CNHDRC, COSAUDE
Yes	28.6%	NCPE-HSE, CONITEC, ACE
***Include patient input in the HTA reports/recommendations***		
Not implemented	67.9%	ICTAHC, DMC, MAHTAS
Assessment report includes patient input	10.7%	NIHTA, HAS, DHTC
HTA recommendations acknowledge or mention the patient input, but no summary is included	1.8%	INFARMED
HTA recommendations include a summary of the patient input	19.6%	NICE, SMC, AOTMIT
***Summarize/inform how patient input was used and impacted the recommendations***		
Not implemented	92.9%	FOPH, IQWIG-G-BA, ZIN
The report with recommendations mentions how patient input was used and impacted the recommendations/decisions	7.1%	NICE, SMC, CDA-AMC

^*^Percentages may exceed 100 % due to the rounding up of numbers.

^**^Providing training, patient participation guidance, a support team for involvement, information about the technology, or a template for submissions.

### 3.3 Ranking of HTA systems and classification into tiers

In addition to establishing a ranking of HTA systems based on their overall score from 0 to 10, four tiers were established to represent the performance of HTA systems worldwide in terms of patient participation. Each HTA body was assigned to a tier based on its overall score, and the tiers are the following: tier 4 (score from 0 to 2.5), Tier 3 (score from 2.5 to 5), Tier 2 (score from 5 to 7.5), and Tier 1 (score from 7.5 to 10). In general, Tier 1 systems have a fully comprehensive patient participation program, Tier 2 systems have strong patient participation programs, Tier 3 systems include patient participation to a certain extent, but have notable room for improvement, and Tier 4 systems have very limited patient participation.

Out of the 56 HTA systems analyzed, two (3.57%) HTA systems were included in Tier 1 with scores of 9.20 and 8.08, respectively, and an average score of 8.64. In Tier 2, the 14 (25%) HTA systems included had an average score of 6.22, and the scores ranged from 5.04 to 7.44. Tier 3 included 22 (39.29%) HTA systems, with scores between 2.56 and 4.94, and an average score of 3.58. Tier 4 comprised 19 (34.5%) HTA systems, with scores ranging from 0.00 to 2.44 and an average score of 1.05. The results and the breakdown by tiers can be found in Table 4. The total set of 56 HTA systems had an average score of 3.61, and 92.86% of all HTA systems analyzed incorporate some sort of patient participation.

**Table 4:** HTA systems ranking and overall score.

**Ranking**	**HTA system**	**Country**	**Score**	**Ranking**	**HTA system**	**Country**	**Score**
1	NICE	United Kingdom	9.20	29	OHA-HERC	United States	3.46
2	CDA-AMC	Canada	8.08	30	HIRA	South Korea	3.26
3	MSAC	Australia	7.44	31	SÚKL	Czech Republic	3.12
4	SMC	United Kingdom	7.34	32	COSAUDE	Brazil	3.06
5	IQWIG-G-BA	Germany	7.12	33	TLV	Sweden	2.88
6	SHTG	United Kingdom	7.04	34	KCE-RIZIV/INAMI	Belgium	2.76
7	PBAC	Australia	6.88	35	WSHCA-HTCC	United States	2.74
8	ICER	United States	6.50	36	IETS	Colombia	2.64
9	DHTC	Denmark	6.34	37	DVSV	Austria	2.60
10	HAS	France	5.98	38	FIMEA	Finland	2.56
11	DMC	Denmark	5.84	39	ACE	Singapore	2.44
12	CATSALUT	Spain	5.50	40	HITAP	Thailand	2.40
13	CONITEC	Brazil	5.46	41	OHTAC	Canada	2.34
14	ZIN	Netherlands	5.32	42	MAHTAS	Malaysia	2.28
15	CONETEC	Argentina	5.24	43	ICTAHC	Israel	2.28
16	NCPE-HSE	Ireland	5.04	44	AGENAS	Italy	1.24
17	PHARMAC	New Zealand	4.94	45	REDETS	Spain	1.20
18	AHRQ	United States	4.76	46	AIFA	Italy	1.00
19	AOTMIT	Poland	4.60	47	RETEHTA	Italy	0.98
20	HTAC	Philipines	4.52	48	DHR-HTAIn	India	0.96
21	NIPHNO	Norway	4.48	49	CRUF	Italy	0.62
22	INESSS	Canada	4.18	50	AEMPS	Spain	0.60
23	NOMA	Norway	4.18	51	HSPI	Vietnam	0.40
24	INFARMED	Portugal	3.70	52	NAMMDR	Romania	0.30
25	NIHTA	Taiwan	3.68	53	CENETEC	Mexico	0.00
26	HIQA	Ireland	3.64	54	CNHDRC	China	0.00
27	FOPH	Switzerland	3.56	55	C2H	Japan	0.00
28	NECA	South Korea	3.56	56	AIHTA	Austria	0.00
Legend	Tier 1	Tier 2	Tier 3	Tier 4			

### 3.4 HTA tier spread across geographies

The performance of the regions regarding the average score of their HTA systems, and its distribution across the four tiers, as well as showing the top two HTA systems per region in terms of average score can be found in Table 5.

**Table 5:** Distribution of HTA systems across tiers per region, average score per region and top 2 HTA systems.

**Region**	**HTAs in tier 1 (%)**	**HTAs in tier 2 (%)**	**HTAs in tier 3 (%)**	**HTAs in tier 4 (%)**	**Average overall score**	**Number of HTAs**	**Top 2 HTAs *(descending order)***
Europe	3.60%	31.00%	37.90%	27.60%	3.75	29	NICE, SMC
Asia	0.00%	0.00%	33.30%	66.70%	2.15	12	HTAC, NIHTA
North America	14.30%	14.30%	57.10%	14.30%	4.58	7	CDA-AMC, ICER
Latin America	0.00%	40.00%	40.00%	20.00%	3.28	5	CONITEC, CONETEC
Oceania	0.00%	66.70%	33.30%	0.00%	6.42	3	MSAC, PBAC

In terms of the distribution of HTA systems per tier across regions, Europe hosted one Tier 1 HTA system, with the majority distributed relatively evenly across Tiers 2, 3, and 4. Asia had some HTA systems in Tier 3, and most were ranked Tier 4. North America housed one Tier 1 HTA system, with the majority falling into Tier 3. In Latin America, most HTA systems were in Tiers 2 and 3. Oceania, boasting the highest average score, had most systems in Tier 2.

### 3.5 Top five HTA systems by phase

The top five HTA systems by phase based on their score per phase can be found in Table 6. In the case that more than one HTA achieved the same score for a certain phase, all those with the qualifying score were included in the same ranking group.

**Table 6:** Top five HTA systems by phase based on their score per phase.

**Ranking**	**Identification and prioritization of health technologies**	**Scoping of assessment**	**Assessment**	**Appraisal**	**Implementation and reporting**
1	NICE, IQWIG–G-BA, HIQA, NOMA, NIPHNO, RETEHTA, CONITEC, CONETEC, HITAP, HTAC	NICE, IETS, HITAP, MSAC, HIQA, CONETEC, DMC, DHTC	IQWIG–G-BA, CONETEC	NICE	NICE
2	SHTG, CDA-AMC, ICER, AHRQ, WSHCA-HTCC, OHA-HERC, ZIN, DHTC, FIMEA, FOPH, TLV, AGENAS, CRUF, CATSALUT, COSAUDE, PBAC, MSAC, NECA, PHARMAC, ICTAHC, HSPI	CDA-AMC, INESSS, ICER, WASHINGTON, OREGON, FOPH, INFARMED, IQWIG–G-BA	MSAC	SHTG	SMC
3	MAHTAS	MAHTAS, REDETS	NIPHNO	ZIN	CDA-AMC
4	N/A	NOMA, NIPHNO, KCE-RIZIV/INAMI, DHR-HTAIn	PBAC	SMC, CDA-AMC	ACE
5	N/A	N/A	NICE, CATSALUT	ICER, AHRQ	CONITEC
Weight per phase	0.4 (4%)	0.8 (8%)	2 (20%)	4 (40%)	1 (10%)

The weight only considers the evaluation of the HTA phases, thus excluding the weight assigned to the variables “capacity building,” “self-evaluation of health technologies” and “geographic scope”; All the HTA systems included in the table involve patients, but the HTA systems included do not represent all the HTA systems involving patients, except for “identification and prioritization of health technologies” and “scoping of assessment,” in which the scoring method only allowed us to distinguish three and four different groups of HTA systems that included patient participation, respectively; Score ranges vary across HTA phases: “identification and prioritization of health technologies” (0.08–0.4), “scoping of assessment” (0.16–0.8), “assessment” (1.04–1.68), “appraisal” (3.28–4), and “implementation and reporting” (0.50–1).

Regarding the phase of identification and prioritization of health technologies, 10 HTA systems shared the highest ranking. For the scoping of assessment phase, eight HTA systems ranked the highest. The assessment phase was led by IQWIG–G-BA and CONETEC. Both the appraisal and implementation and reporting phases were led by NICE.

### 3.6 Occurrence of patient participation by tier and phase

**Figure 4:**
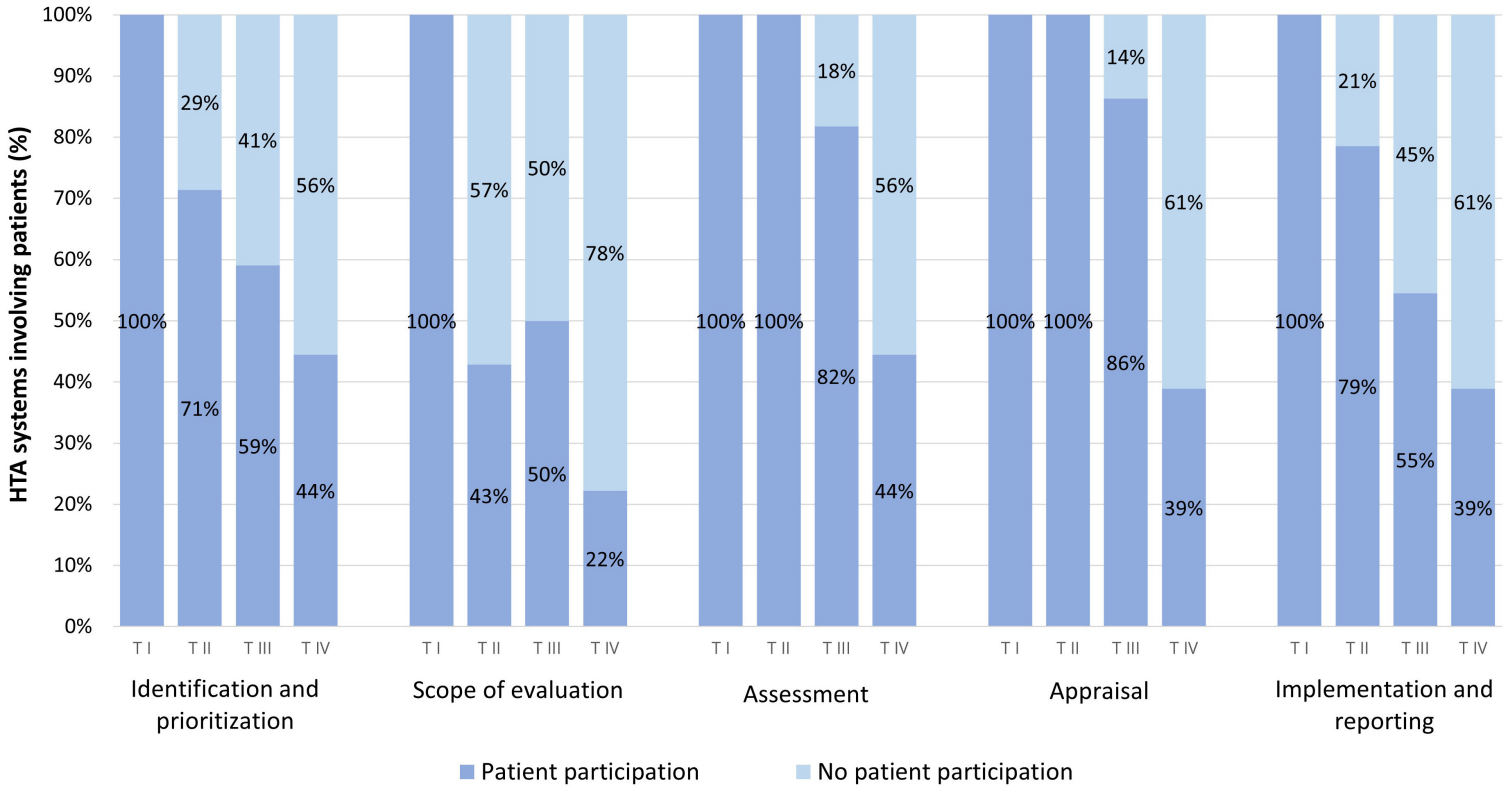
Occurrence of patient participation by phase and tier.

The occurrence of patient participation (without considering the depth of participation per variable) by tier and phase can be found in Figure 4. All HTA systems in Tier 1 involve patients across the five HTA phases. In Tier 2 and 3 HTA systems, the occurrence of patient participation is similar in different phases, and is most common during assessment and appraisal, while it is least common in the scope of evaluation phase. Regarding Tier 4 HTA systems, the occurrence of patient participation is below 50% for all phases, and the scope of evaluation phase hosts the least occurrence.

### 3.7 Occurrence of patient participation by tier and variable

**Figure 5:**
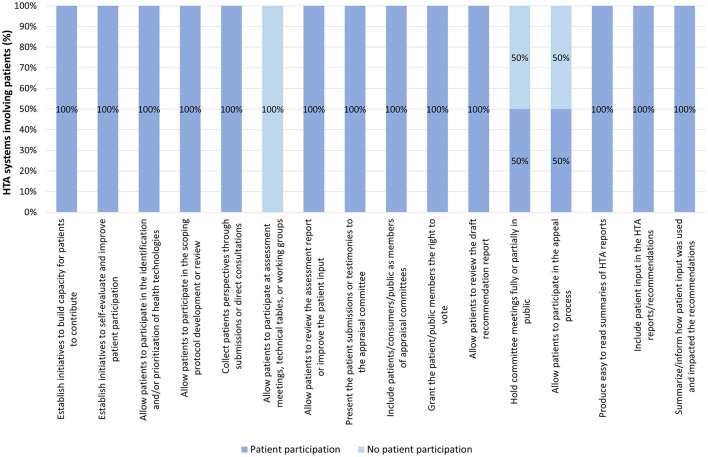
Occurrence of patient participation per variable across Tier 1 HTA systems.

Regarding the occurrence of patient participation by tier and variable, Tier 1 HTA systems involved patients in all but three variables (excluding the variable “geographic scope of the HTA system”), without considering the depth of participation per variable. Neither of the Tier 1 HTA systems allowed patients to participate at assessment meetings, technical tables, or working groups during assessment. Additionally, CDA-AMC did not hold committee meetings in public, nor did it allow patients to participate in the appeal process. Further details can be found in Figure 5.

**Figure 6:**
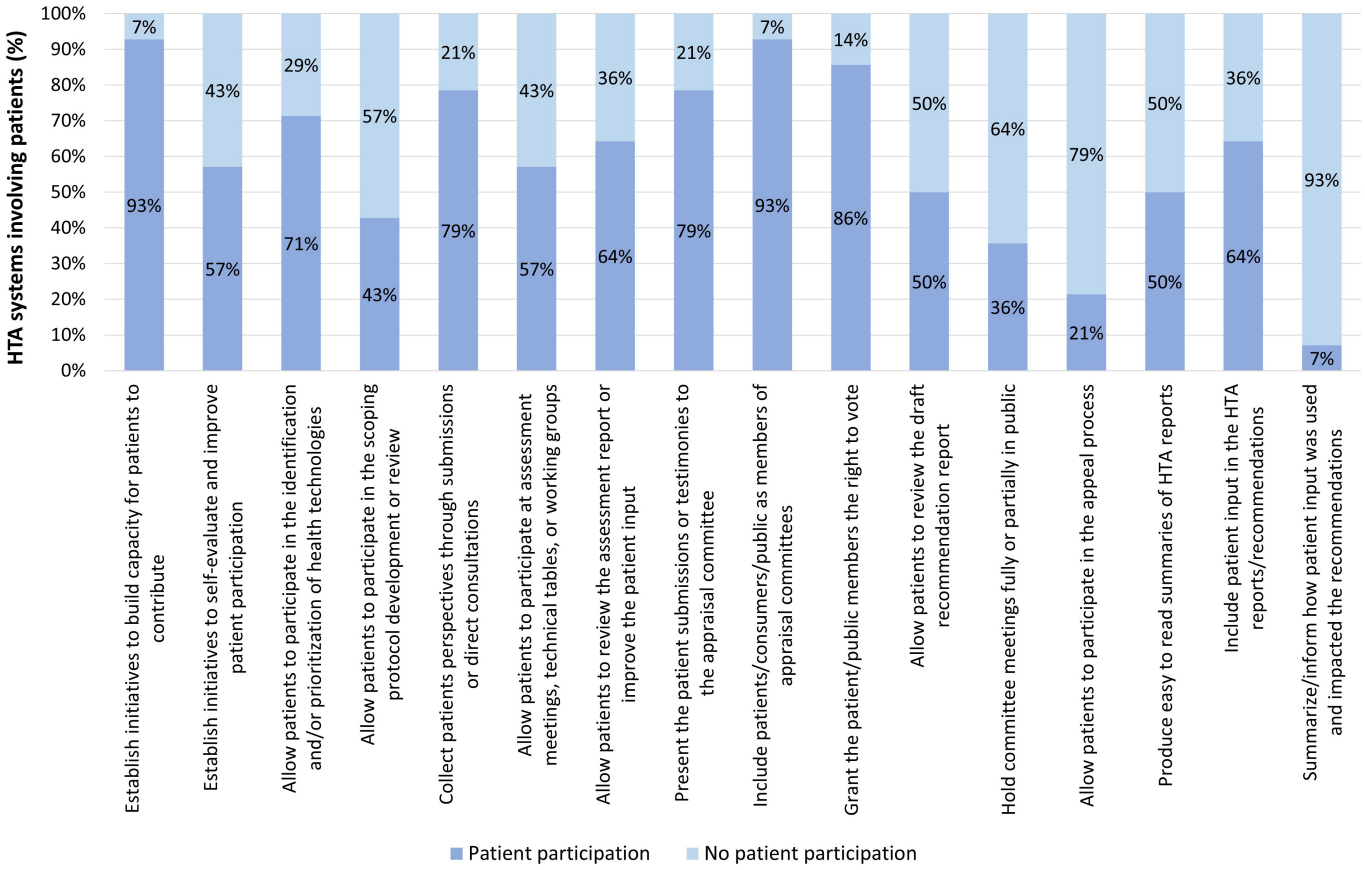
Occurrence of patient participation per variable across Tier 2 HTA systems.

In Tier 2 systems, the three variables with the highest occurrence were, in descending order: building capacity for patients to contribute and including patients/consumers/public as members of appraisal committees (93% for both), and granting them the right to vote (86%). Further details can be found in Figure 6.

**Figure 7:**
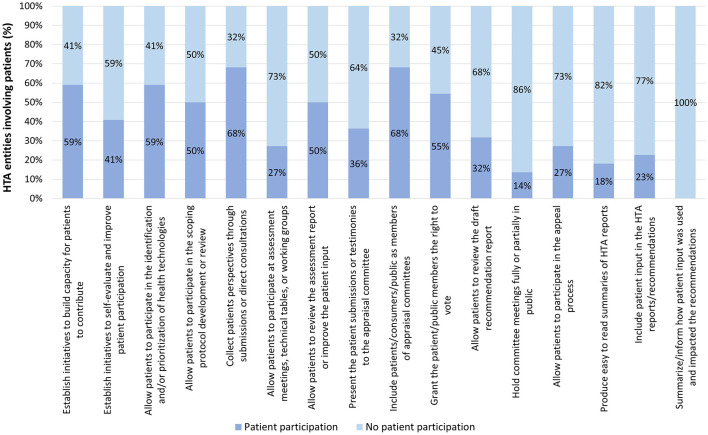
Occurrence of patient participation per variable across Tier 3 HTA systems.

For those systems in Tier 3, the three variables with the highest occurrence were, in descending order: collecting patients' perspectives through patient submissions or direct consultation and including patients/consumers/public as members of appraisal committees (68% for both), building capacity for patients to contribute, and allowing patients to participate in the identification and/or prioritization of health technologies for evaluation (59% for both). Further details can be found in Figure 7.

**Figure 8:**
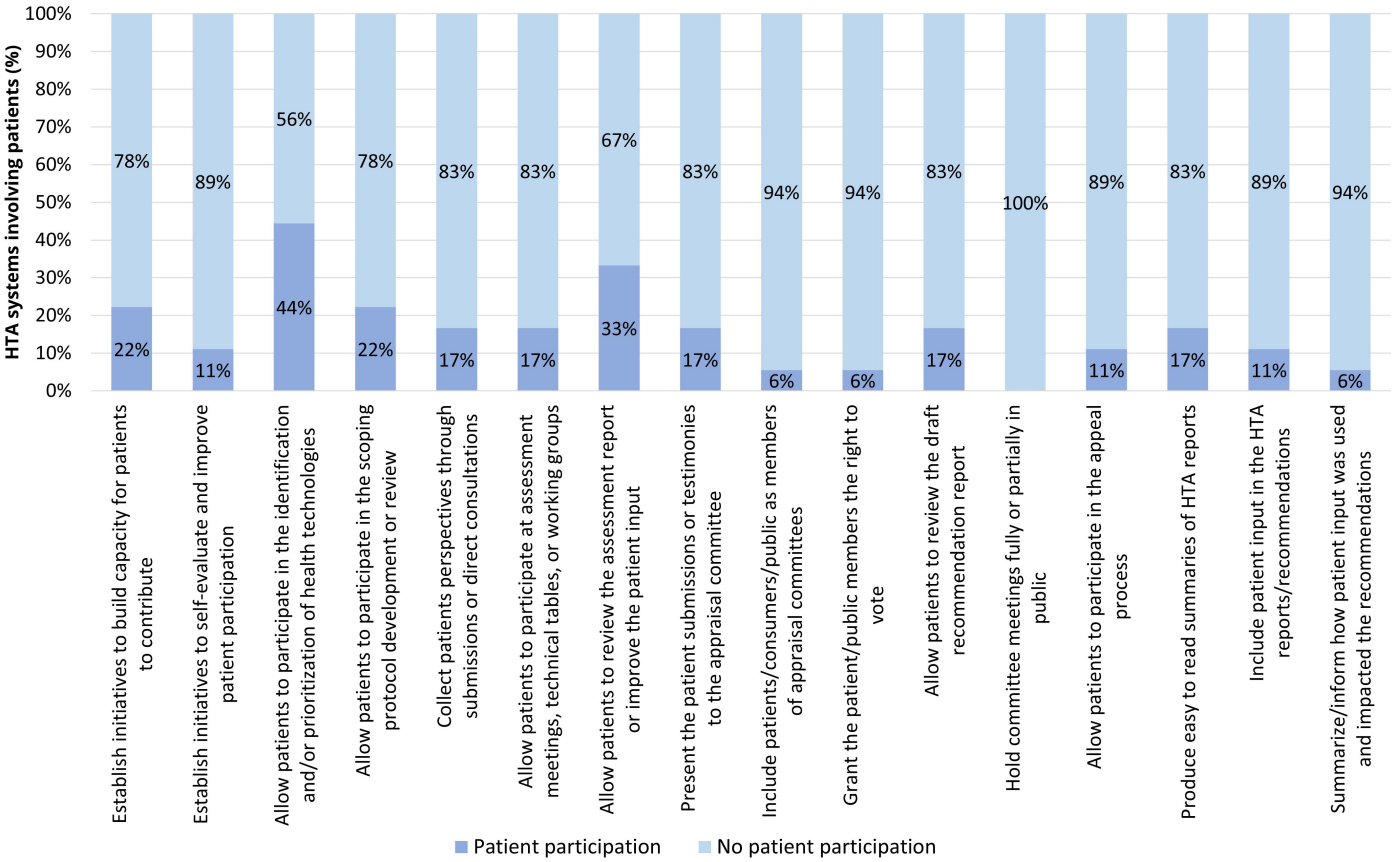
Occurrence of patient participation per variable across Tier 4 HTA systems.

Finally, in Tier 4 systems, the three variables with the highest occurrence were, in descending order: allowing patients to participate in the identification and/or prioritization of health technologies (44%), allowing patients to review the assessment report or improve the patient input (33%), building capacity for patients to contribute and allowing patients to participate in the scoping protocol development or review (22% for both), and collecting patients' perspectives through patient submissions or direct consultation, allowing patients to participate at assessment meeting, technical tables or working groups, presenting the patient submissions or testimonies to the appraisal committee, allowing patients to review the draft recommendations report, and producing easy to read summaries of HTA reports (17% for all of them). Further details can be found in Figure 8.

## 4 Discussion

While previous articles have described and compared patient participation within HTA systems (6, 17, 19–26), to our knowledge, this publication, though limited to publicly available information, represents the first comprehensive, structured, comparative review and ranking of HTA systems worldwide focusing on the breadth and depth of patient participation in their HTA processes.

There are prior attempts to classify patient participation in HTA (18). The framework developed by Gauvin, for example, emphasized the type of participant, the stage of the HTA process, and the levels of involvement, while the range of specific participation mechanisms received less attention. In contrast, our framework placed priority on these mechanisms (e.g., submission of input, patient membership, voting rights), as we considered them central to understanding how participation is operationalized. While inspired by existing models and comparisons, we therefore developed a new framework tailored to this objective. In addition, our evaluation incorporated a quantitative scoring approach. Such measures can facilitate standardized cross-country comparisons, enhance HTA processes, promote accountability and transparency, empower patient advocacy groups to argue for stronger involvement, and support evidence- based policymaking.

In the context of our ranking of the HTA systems, the two systems in Tier 1 (NICE in England and Wales and CDA-AMC in Canada) and some Tier 2 HTA systems (e.g., MSAC and PBAC in Australia, SMC in Scotland, IQWIG and G-BA in Germany) have previously garnered recognition for their substantial efforts in integrating patient participation throughout their HTA processes (6, 11, 15, 17, 25). This reaffirms their status as exemplary cases of patient participation within HTA systems. In contrast, other Tier 2 ranked systems, such as HAS in France, DMC and DHTC in Denmark, or ICER in the United States, represent more recent developments in terms of patient participation. In general, best practice examples are characterized by a substantial breadth and depth of patient participation throughout the HTA process, utilizing a variety of participation mechanisms and leveraging them meaningfully. These HTA systems notably engage the patient community, either in conjunction with or distinct from the broader public or citizenry.

It is worth noting that the top performing HTA systems overall may not excel in all phases. For example, NICE, while ranked 1st overall, falls to the 5th group in the assessment phase–a category led by G-BA (ranked 5th overall) and CONETEC (ranked 16th overall) in Argentina. This could be attributed to various factors, such as the diversity of HTA processes and the resulting opportunities for participation in each phase.

Our results show that 92.86% of all HTA systems analyzed incorporate some sort of patient and/or public participation, a higher figure than previously reported studies, which range from 50 to 81% (32–34). This discrepancy may be due to our inclusion of both patient and public participation, as well as participation in HTAs for both pharmaceuticals and medical devices, whereas previous studies often separated these categories, possibly resulting in lower figures. Other factors such as the increasing trend of patient participation in HTA (4, 7), differences in the sample of HTA systems analyzed, or the variables used to assess participation, may also explain this difference. However, despite this high participation rate, when considering that only 28.57% fall into Tier 1 or Tier 2, and that the average score for the entire set of HTA systems is 3.61, there appears to be substantial room for global improvement in the depth of patient participation, as highlighted in the literature (4, 7, 8). This gap between the breadth and depth of patient participation might be explained by several reasons, such as HTA systems still considering patient participation as an optional extra rather than a vital component and considering patient participation as a “token” activity (7). The variability in patient participation among HTA systems can be attributed to multiple causes. For instance, some articles have underscored barriers to patient participation in HTA, such as the need for resources, limited patient understanding of the HTA process, administrative burdens and delays, and a lack of knowledge on how to effectively utilize patient input (6, 17). Additionally, the specific context of individual countries, including national administrative traditions and the characteristics of healthcare and HTA systems, has been identified as a challenge to the transferability of patient participation practices and guidelines from one country to another (5, 35). Finally, given that HTA is relatively recent in certain regions such as in Asia or Africa (36, 37), the time between the establishment of the HTA system and the initiation of patient involvement may also impact the degree of patient participation, necessitating further investigation.

In terms of patient participation across the phases of the HTA process, our results, indicating that 19.6% of HTAs involve the patient community in all phases, regardless of the level of participation, align to some extent with international comparisons (34). That study also underscores patient participation at every phase of HTA, but discrepancies in methodologies used by this and other articles hinder a direct comparison of the results with our research. The elevated occurrence of participation during the assessment and appraisal phases, as identified in our research, may be attributed to factors such as the perceived significance of these phases in the overall HTA process, a tendency to involve patients later in the evaluation process, increased opportunities for participation, or a tendency to engage patients when recommendations are made. Nonetheless, further exploration and standardization of methodologies in future studies could contribute to a more comprehensive understanding of patient participation across phases.

According to our findings and supported by previous studies (17, 19–21), patient participation is incorporated through a variety of mechanisms during the HTA process, one of the most common being the participation of the public or patients as members of appraisal committees. This might be indicative of the increasing level of patient participation, given the recognized relevance of this mode of participation (17). In contrast, the least common mechanism of participation is reporting how patient input was used and impacted HTA recommendations. This finding aligns with previous studies (4, 6, 22, 38), suggesting that, despite the evident trend toward involving the patient community, the actual influence of patients on decision-making remains unclear.

When analyzing HTA systems by region and their distribution in tiers and average scores, it should be noted that the varying number of systems assessed per region can make it challenging to draw broad conclusions.

Although Oceania had the highest average score, only three systems were analyzed. The MSAC and PBAC in Australia are noteworthy examples of effective patient and public involvement in HTA. Ranked 3rd and 7th, respectively, these systems actively engage the consumer community throughout the HTA process. They allow consumer nominations for technology evaluation, invite input through open calls, include consumers as members in assessment teams and appraisal committees, and consumer input is summarized in public documents, demonstrating a commitment to transparency and inclusivity. In the scoping phase, MSAC takes an additional step by including a consumer member in the scoping team.

In North America, which had the second highest average, a few national systems stand out, while the regional systems analyzed involve the patient community to a lesser but growing degree. CDA-AMC, ranked 2nd, showcases a comprehensive commitment to engaging the public and, notably, the patient community throughout the entire HTA process (6). Patient groups are encouraged to contribute information to submissions early in the HTA process, and patient representatives serve as members on appraisal committees. CDA-AMC extends support through guidance, training, travel awards, and access to a specialized team, and regularly evaluates and refines its processes, placing significant value on the feedback provided by stakeholders. An example of an emerging case is ICER in the US, ranked 8th. In 2020, ICER initiated a Patient Engagement Program, and although it is of recent creation, it already reflects a strong commitment to patient participation within its framework (39).

In Europe, the larger number of systems assessed might explain the even distribution across tiers, coming in 3rd place in terms of average score. Notably, NICE and SMC, secured the 1st and 4th rankings, respectively, showcasing exemplary systems. NICE is globally recognized for the comprehensive involvement of the public and patient community throughout the entire HTA process. It incorporates diverse perspectives, offers extensive support through its Patient Participation Program, and actively seeks feedback and implements self-evaluation strategies to continuously improve patient participation. Similarly, SMC actively encourages patient contribution, facilitating participation through a dedicated Public Participation Team, submission templates, and a user-friendly website. Improvements have been seen in certain HTA systems, such as HAS (ranked 10th), DHTC and DMC (ranked 9th and 11th), or KCE-INAMI/RIZIV in Belgium (ranked 33rd). HAS, for example, began involving the patient community in 2016, developing an online contribution process, including patient representatives as voting members in appraisal committees, and publicly disclosing a comprehensive self-evaluation of their user/citizen participation methods. In contrast, AIHTA in Austria, despite having expressed intentions to involve patients, it showed limited evidence of patient participation (40). Importantly, HTA in Europe is undergoing significant changes toward more unified market access, particularly through the introduction of the Joint Clinical Assessment (JCA). JCA will centralize the clinical evidence assessment phase at the European level, and consequently, patient involvement in this phase will be coordinated at the European level. However, since the appraisal phase will remain the responsibility of individual countries, patient involvement in this phase will vary depending on national policies. This evolving framework highlights the importance of updating our study to align with these changes.

Latin America, where only a few HTA systems were analyzed, placed fourth in terms of average score. As reflected in both our study results and previous literature (41), although patient and public participation is generally lacking, notable examples exist, including CONITEC (ranked 13th) in Brazil and CONETEC (ranked 15th) in Argentina. CONITEC, recognized in the literature (41–43), allows technology nominations, patient submissions, and participation during appraisal committee meetings. However, testimonials suggest that patient participation may be insufficient (44). CONETEC also firmly incorporates patient participation, especially in the assessment phase, including an open call for disease experience submissions, a patient panel guiding technical teams, or appointing patient representatives in technical teams. In Colombia, while patients play a significant role in the development of clinical guidelines by the IETS, patient participation in HTA is less prominent (41, 45).

In Asia, HTA adoption has been historically low, and has grown considerably over the last two decades, which may contribute to the current scarcity of patient participation, as indicated by both average scores and the HTA systems distribution across tiers (36). Two cases that incorporate patient participation are the HSPI in the Philippines and NIHTA in Taiwan, ranked 20th and 25th, respectively. HSPI includes patient and public participation primarily through consultations conducted throughout the HTA process. Moreover, a citizen's representative holds a voting position in the appraisal committee. NIHTA conducts an open call for information submissions by patients and patient groups, and patient representatives can participate in committee meetings (6). In Thailand (ranked 40th), while HITAP initiated patient participation as early as 2009 and introduced patient participation guidance in 2012 (6, 46), its ranking reflects our understanding that implementation is limited. Currently, patients participate in the identification and prioritization phase and provide input during the scoping and assessment phases. Although HTA agencies in Asia are relatively new, HTA is gaining recognition. Moreover, valuable initiatives have emerged, notably HTAsiaLink, a network established in 2011 to strengthen HTA capacity in Asia, with patient involvement being one of their topics of discussion. Consequently, we anticipate a growth in patient participation (47).

A key lesson from higher-ranked HTA systems is that strong patient participation requires both breadth and depth: mechanisms must be available across all phases of the HTA process, and they must go beyond symbolic consultation to provide patients with structured, influential roles. Exemplary systems, such as NICE in the United Kingdom, or CDA-AMC in Canada, combine multiple layers of engagement, from early identification of topics to voting rights on appraisal committees, supported by dedicated resources such as patient engagement teams, training programs, and feedback mechanisms that show how patient input was used. By contrast, lower-ranked systems often limit participation to later phases or to isolated mechanisms (e.g., open consultations without follow-up), which risks tokenism and undermines trust. For policymakers, the implication is clear: institutionalizing patient participation effectively requires embedding participation structurally (e.g., committee membership, voting rights), procedurally (e.g., systematic calls for input, dedicated capacity-building for patient groups), and in terms of transparency (e.g., public reporting on how input influenced recommendations).

In this study, the term “patient participation” was used broadly to include both patients and the general public. While public involvement can sometimes capture elements of the patient voice, it is not equivalent to direct patient input, and this approach may overestimate the true extent of patient participation. For example, the inclusion of citizen representatives on committees or open public consultations does not necessarily ensure that the lived experiences and specific needs of patients with the condition under evaluation are represented. We adopted this inclusive definition to avoid overlooking mechanisms that contribute to legitimacy and transparency, but acknowledge that future research should distinguish more clearly between patient and public involvement.

Future research should incorporate structured validation with local experts and stakeholders, and explore consensus methods such as Delphi panels to refine weighting schemes. This would increase reproducibility and alignment with international initiatives on patient involvement in HTA.

## 5 Conclusion

In conclusion, this paper represents a significant effort in the field of international HTA by offering a structured and comparative review of HTA systems worldwide, focusing on patient participation. Patient participation in HTA is crucial, as it brings valuable perspectives to the forefront, making healthcare coverage decisions more patient-centered and equitable.

Our findings reveal variety in practices across regions and tiers, with some systems setting examples in patient participation, while others are on a journey of development, highlighting room for improvement globally.

While our study found that most HTA systems include some level of patient or public participation, the average scores across countries and their distribution in ranking tiers suggest a generally low depth of patient involvement in these programs worldwide. As HTA evolves, we anticipate greater patient impact and a more even distribution of systems across ranking tiers.

This study serves as a foundation for further research and as a point of reference for HTA systems looking to strengthen their patient engagement initiatives. Continuous improvement is key to ensuring that HTA processes worldwide are not only rigorous but also patient-centered, ultimately contributing to more informed, fair, and patient-focused healthcare decisions.

Considering that both HTA and patient participation in it are gaining recognition worldwide, updating our study accordingly would be beneficial.

## 6 Limitations

This research has certain limitations that could influence the comprehensiveness of the data and scoring for HTA systems. Chief among these was that data collection relied exclusively on secondary research, with no primary research or systematic surveys conducted. The availability and detail of information vary across systems, potentially leading to over- or under-representation due to publication bias. This raises concerns about accessibility, as experts might struggle to find data, indicating potential challenges for the general public and patients. Moreover, because the study relied exclusively on desk research, the findings have not been verified with HTA bodies or local experts. Such validation would be important in future work to reduce the risk of omissions or misclassifications and to strengthen the robustness of comparisons across systems.

Language barriers also posed a limitation, with non-English literature likely under-represented in databases like PubMed. The ability of the study team to conduct searches in local languages or access grey literature was limited.

There is also a significant degree of heterogeneity in the structure and processes of the HTA systems analyzed in this study. In particular, it was sometimes difficult to partition the HTA process into distinct phases, mapping the procedures of specific bodies to these phases, and identifying the various bodies comprising an HTA system, particularly in cases where more than one body is involved.

In addition, the scoring system did not distinguish between a lack of patient participation and a lack of information on patient participation. Additionally, the scoring system considered the frequency of patient participation, but inconsistencies in reporting could have influenced scores. It was sometimes unclear whether patient participation was representative of typical practice or limited to specific assessments.

Distinguishing between implemented and planned patient participation was another challenge, potentially affecting the accuracy of our assessment. Finally, there is no consensus on measuring patient participation in HTA, and our methodology represents just one perspective, with the variables used not encompassing all possible forms of involvement.

An additional limitation lies in the subjectivity of the weighting system applied to participation activities. While conceptually grounded in the depth, influence, and transparency of each activity, the assignment of Low, Medium, High, or Very High weights was not externally validated (for instance through a Delphi panel or stakeholder consultation). This introduces an element of judgement that could affect reproducibility if applied by other reviewers.

Finally, our inclusive definition of patient participation, which also encompassed broader public involvement, may have inflated estimates of actual patient input.

## Data Availability

The original contributions presented in the study are included in the article/Supplementary material, further inquiries can be directed to the corresponding authors.
